
JOURNAL INFORMATION
==============================
NLM Title Abbreviation: J High Energy Phys
Journal ID: J High Energy Phys
NLM Journal ID: 101668727

Journal of High Energy Physics

ISSN: 1126-6708
EISSN: 1029-8479

ARTICLE INFORMATION
==============================
PMCID: PMC8827410
PMID: 35212686
DOI: 10.1007/JHEP02(2022)031
Article ID: 17716
Article version: 1
Subjects: Erratum

Erratum to: MiNNLOPS: a new method to match NNLO QCD to parton showers

Monni Pier Francesco pier.monni@cern.ch
1
Nason Paolo paolo.nason@mib.infn.it
2
Re Emanuele emanuele.re@lapth.cnrs.fr
1 3
http://orcid.org/0000-0002-3527-3653 Wiesemann Marius marius.wiesemann@cern.ch
1 4
Zanderighi Giulia zanderi@mpp.mpg.de
4
1 grid.9132.9 0000 0001 2156 142X CERN, Theoretical Physics Department, CH-1211 Geneva 23, Switzerland
2 grid.4708.b 0000 0004 1757 2822 Università di Milano - Bicocca and INFN, Sezione di Milano – Bicocca, Piazza della Scienza 3, 20126 Milano, Italy
3 grid.462959.5 0000 0001 2224 4709 LAPTh, Université Grenoble Alpes, Université Savoie Mont Blanc, CNRS, 74940 Annecy, France
4 grid.435824.c 0000 0001 2375 0603 Max-Planck-Institut für Physik, Föhringer Ring 6, 80805 München, Germany
Electronic publication date: 2022 Feb 4
Print publication date: 2022
Volume: 2022
Issue: 2
Electronic Location ID: 31
Received 2022 Jan 10; Accepted 2022 Jan 11
Copyright: © The Author(s) 2022
License: Open Access. This article is distributed under the terms of the Creative Commons Attribution License (CC-BY 4.0), which permits any use, distribution and reproduction in any medium, provided the original author(s) and source are credited.
License URL: https://creativecommons.org/licenses/by/4.0/

Erratum for: 10.1007/JHEP05(2020)143
issue-copyright-statement: © The Author(s) 2022

==============================
Erratum to: JHEP05(2020)143

ArXiv ePrint: 1908.06987

The online version of the original article can be found at 10.1007/JHEP05(2020)143

Publisher’s Note

Springer Nature remains neutral with regard to jurisdictional claims in published maps and institutional affiliations.


Reference

[1] Monni PF Nason P Re E Wiesemann M Zanderighi G MiNNLOPS: a new method to match NNLO QCD to parton showers JHEP 2020 05 143 10.1007/JHEP05(2020)143 PMC8827410 35212686
